# Cultural Differences in Humor Perception, Usage, and Implications

**DOI:** 10.3389/fpsyg.2019.00123

**Published:** 2019-01-29

**Authors:** Tonglin Jiang, Hao Li, Yubo Hou

**Affiliations:** ^1^Department of Psychology, University of Hong Kong, Pok Fu Lam Hong Kong; ^2^School of Psychological and Cognitive Sciences, Peking University, Beijing, China; ^3^School of Psychological and Cognitive Sciences and Beijing Key Laboratory of Behavior and Mental Health, Peking University, Beijing, China

**Keywords:** humor, humor perception, humor usage, psychological wellbeing, cultural difference, Eastern, Western

## Abstract

Humor is a universal phenomenon but is also culturally tinted. In this article, we reviewed the existing research that investigates how culture impacts individuals’ humor perception and usage as well as humor’s implications for psychological well-being. Previous research has substantiated evidence that Easterners do not hold as positive an attitude toward humor as their Western counterparts do. This perception makes Easterners less likely to use humor as a coping strategy in comparison with Westerners. Despite this difference, Westerners and Easterners have similar patterns in the relationship between their humor and psychological well-being index, though the strength of the relationship varies across cultures. Implications and potential future research avenues discussed.

## Cultural Differences in Humor Perception, Usage, and Implications

Humor refers to the tendency to experience or express what is amusing and funny, which is always accompanied with emotional response and vocal-behavioral expressions, such as laughter and smiling (Chen and Martin, 2007; Martin and Ford, 2018). Generally, humor is present in all human cultures (Fry, 1994). However, people from different cultural backgrounds may see humor in different ways. As it is remarked by Martin and Ford (2018):

Humor is a universal human activity that most people experience many times over the course of a typical day and in all sorts of social contexts. At the same time, there are obviously important cultural influences on the way humor is used and the situations that are considered appropriate for laughter (p. 30).

Humor is universal but also culturally specific. Previous literature has shown that Easterners and Westerners differ in humor perception (e.g., Chen and Martin, 2005, 2007), however, the results about East-West cultural difference in humor usage, the relationship between humor and psychological well-being are rather mixed and inconsistent (e.g., Kazarian and Martin, 2004; Chen and Martin, 2007; Hiranandani and Yue, 2014). Whether there is East-West cultural difference in humor perception, usage, as well as humor and psychological well-being relationship remains unclear. Understanding how culture influences humor perception, humor usage, as well as humor’s implications for psychological well-being is of great importance because humor has significant consequences for human psychological well-being (e.g., Martin, 2001; Chen and Martin, 2007; Martin and Ford, 2018). To clarify this question, we need to have a systematic view about cultural differences in humor. The aim of this article provides a review of how culture influences humor perception and usage as well as the relationship between humor and psychological well-being so as to shed light on the issue of using humor to promote individuals’ psychological well-being. Lastly, we also suggest some revenues for future research.

## Cultural Differences in Humor Perception

Since the era of Ancient Greece, it has been a long tradition among Westerners to embrace humor (Grant, 1924/1970; Martin and Ford, 2018). Westerners have associated humor with positivity and seen humor as natural amusement expressions (Apte, 1985). In the late nineteenth century, Freud (1928) regarded humor as a defense mechanism against obstacles and distress. In the perspective of Psychoanalysis, humor was regarded not only as a way to help people release fear and anxiety, but it also provides an amusing, funny, and less scaring perspective toward people’s inner fear (Martin and Ford, 2018). In the twentieth century, psychologists in Western world began to talk about the positive effects of humor. For example, humor is regarded as a desirable positive trait of an individual (Allport, 1937; Maslow, 1968; Mintz, 1983; Mindess et al., 1985). Humorous people are thought to be more attractive (e.g., Regan and Joshi, 2003; Fraley and Aron, 2004) and more motivating, creative, and capable (e.g., Sternberg, 1985; Priest and Swain, 2002). Humor also takes on meaning as an essential element of psychological health associated with self-awareness, well-adjustment, and affability (Allport, 1961; Martin and Ford, 2018).

In sharp contrast, Easterners’ attitudes toward humor are not that positive. Specifically, in China, Confucianism has devalued humor. Chinese self-actualization denigrates humor while stressing restriction and seriousness (Bond, 1996; Liao, 1998, 2007; Yue, 2010). Chinese are reluctant to admit they are humorous out of fear of jeopardizing their social status. Chinese do not think that humor is a desirable personality trait (Rudowicz and Yue, 2002; Yue, 2011).

The views toward humor are reflected in behaviors. Surveys and empirical research provide evidence for the cultural differences in humor perception. For example, Chinese do not see humor as an essential element of creativity (e.g., Rudowicz and Yue, 2011; Yue and Hui, 2011, 2015) like Westerners do (e.g., Murdock and Ganim, 1993; Kellner and Benedek, 2017; Lu et al., 2019). Yue X. et al. (2016) found that Canadians rated humor more importantly than their Chinese counterparts. They also provided evidence for the assumption that Easterners do not associate humor with positivity as Westerners do by finding that Hong Kong participants who were primed with Western culture would like to use more positive words to depict a humorous person than participants who were primed with Chinese cultural icons. Moreover, Chen and Martin (2005) found that Chinese students rated themselves as being less humorous than Canadian students. Yue X. et al. (2016) also found similar results. East Asians, including mainland Chinese, Taiwanese, and Hong Kong people, were consistently found to report lower self-rated humor than Westerners (e.g., Liao, 2001; Chen and Martin, 2005, 2007; Liao and Chang, 2006). This research provides direct evidence that Westerners associate humor with positivity, which is not the case for Chinese.

However, Chinese culture is not purely dominated by Confucianism. Other philosophies also impact Chinese attitudes toward humor (Yue, 2010, 2011). One of these philosophies is Taoism. Different from Confucianism’s despising on humor, Taoism regards humor as “an attempt of having witty, peaceful and harmonious interaction with nature” (Yue, 2011, line 4, p. 464). The tug between different philosophies makes the appreciation–despising complex toward humor deeply rooted in Chinese culture, which makes Chinese have quite an ambivalent attitude toward humor (Yue, 2010, 2011). Yue (2011) summarized three ambivalent attitudes toward humor among Chinese. The first is valuing humor but considering themselves to lack the trait of humor. The second is how being humorous is not associated with being an orthodox Chinese. The third is that humor is not important for everyone but exclusively for those with expertise. He also conducted survey research to test his assumptions, which showed that even though Chinese thought that humor was important in daily life, they would not say that they were humorous themselves. When asked to choose personality attributes, the top 10 important personality attributes that Chinese chose for humor were fundamentally different from those they chose for the Chinese personality. Chinese were likely to nominate a person with humorous expertise rather than an ordinary person as being a humorous person (Yue, 2011). Other research evidence supports this assumption as well. As Yue X. et al. (2016) found in the humor nomination study, Chinese prefer to name experts rather than their friends or relatives as humorous people, whereas the opposite was true for Canadians. Similar results were also found by Yue et al. (2010b) and Yue and Hiranandani (2014). Moreover, using the Implicit Association Test (IAT), Jiang et al. (2011) found that despite Chinese students not showing significant differences in explicit attitudes toward humor in comparison with American students, they were more likely to associate humor implicitly with negative adjectives.

In summary, Westerners and Easterners’ views toward humor fundamentally differ from each other. Westerners regard humor as a desirable trait of an ideal self, associate humor with positivity, and stress the importance of humor in their daily life. On the contrary, Easterners’ attitudes toward humor are not that positive. Specifically, the apprehension–despising complex makes Chinese have ambivalent attitudes toward humor. Even though Chinese might sometimes admit that humor is important in daily life, they do not think they are humorous themselves. For Chinese, humor is a talent that exclusively belongs to experts and is not a desirable trait of their ideal personality.

## Cultural Difference in Humor Usage

Cultural difference in humor perception directly influences humor usage. In Western culture, humor has become an indispensable coping strategy for Westerners (Moran and Massam, 1999; Lefcourt, 2001). According to psychoanalysis theory, humor acts as a defense mechanism to help people fight against negative events (Freud, 1928; Shurcliff, 1968). On the one hand, humor serves as a catharsis for negative energy (Freud, 1960/1905). On the other hand, humor allows people to perceive the anger and fear arising from incongruity in different ways (Martin and Ford, 2018). However, the appreciation–despising complex makes things different in Chinese culture. As we discussed above, Westerners tend to regard humor as a common positive trait, whereas Chinese tend to see humor as a special talent that is not commonly seen in ordinary people. Thus, it is unsurprising that Westerners tend to use humor more frequently than Chinese (e.g., Liao, 2001; Chen and Martin, 2005, 2007; Liao and Chang, 2006; Yue, 2011). Research has found a West-East cultural difference in humor usage. For example, humor is not an important coping device in Japan as it is in the United States (Abe, 1994). Chinese students were less likely to use humor as a coping strategy with stress than their Canadian counterparts (e.g., Chen and Martin, 2005, 2007). Similarly, Singaporean students were less likely to use humor to cope with difficulty than American students (Nevo et al., 2001). As suggested by Yue X. et al. (2016), in Western countries, no matter who they are, people have a general tendency to use humor in coping with stress or difficulties, which is not true in Eastern countries.

To be specific regarding cultural differences in humor usage, we turn to the four types of humor: self-enhancing, affiliative, self-defeating, and aggressive humor (Martin et al., 2003). The four types of humor have been found to be applicable in different countries, such as Canada, China, the United States, Lebanon, and Belgium (Saroglou and Scariot, 2002; Martin et al., 2003; Chen and Martin, 2007; Taher et al., 2008). However, people from different cultural backgrounds may use them in different ways.

Research on usage differences in the four types of humor across countries or regions indicates that Easterners tend to use more adaptive humor, while Westerners tend to use more maladaptive humor. For example, Hiranandani and Yue (2014) found that students from India and Hong Kong, both having cultures prizing collectivism, used more affiliative and self-enhancing humor than aggressive and self-defeating humor. Similarly, Hong Kong students reported more use of aggressive and self-defeating humor and less use of affiliative and self-enhancing humor than mainland Chinese students (e.g., Yue et al., 2010a, 2014b; Yue X. D. et al., 2016). This could be explained by the fact that the bicultural background of Hong Kong makes Confucianism and collectivism less influential there than in mainland China.

In addition, Chen and Martin (2007) asked Chinese and Canadian students to complete the Humor Styles Questionnaire (HSQ) and Coping Humor Scale (CHS). They found that Canadian students reported using the four types of humor more than their Chinese counterparts did, especially aggressive humor. No significant relationship was found between aggressive humor and coping in Chinese students, indicating that Chinese are less likely to use aggressive humor as a coping strategy. This difference is unsurprising. Aggressive humor is associated with high individualism, which emphasizes independence and assertiveness, and low collectivism, which de-emphasizes interdependence and harmony (Kazarian and Martin, 2006; Martin and Ford, 2018). Even as children, Chinese tend to see humor as a sign of aggression and as disruptive to social relationships, whereas Canadians tend to see humor as a socially desirable leadership trait (Chen et al., 1992).

Additionally, Kalliny et al. (2006) found differences in Arab and American humor usage, specifically that Americans used significantly more self-defeating and self-enhancing humor, while there were no differences in the use of affiliative and aggressive humor. They suggested that the greater use of self-defeating humor in American culture may be due to the desire to equalize and lower power distances present in American culture, while greater use of self-enhancing humor may be attributed to the fact that self-enhancing humor helps them gain focus and attention (Kalliny et al., 2006).

These studies were conducted to examine cultural differences in humor usage across countries without taking specific cultural variables into consideration. Thus, it is still not quite clear whether individual or cultural differences account for the differences in humor usage.

Kazarian and Martin (2004) systematically investigated the relationships between culture and the four types of humor usage in terms of specific cultural dimensions. Comparing Lebanese, Canadians,’ and Belgians’ humor usage, they found that individuals from horizontal collectivist cultures that emphasize harmony and group cohesion are more likely to use affiliative humor, whereas individuals from a vertical collectivist culture that values self-sacrifice for the sake of group are more likely to employ self-defeating humor. Furthermore, individuals from vertical individualist cultures that embrace competitiveness are more likely to use aggressive humor to enhance their hierarchical status. It was also found that affiliative humor was used to the same degree among the various cultural orientations (Kazarian and Martin, 2004). This research helps to clarify how culture impacts humor usage. However, a survey only provides correlational evidence; more refinement is needed.

Moreover, specific humor types may have different connotations across cultures, which would influence humor usage in different cultural backgrounds. For example, Chen and Martin (2007) found that Chinese considered the item “If I am having problems or feeling unhappy, I often cover it up by joking around, so that even my closest friends don’t know how I really feel” to be more like self-enhancing humor than self-defeating humor, like Canadians did. This indicates that using humor to conceal one’s problems is more of a self-enhancing than a self-defeating strategy for Chinese (Chen and Martin, 2007). This may also be attributed to the fact that saving face is one of the most important attributes for the Chinese personality (e.g., Gao, 1998; Yue, 2011). Thus, one would expect people to use humor according to its culturally tinted connotations and thus use humor differently.

In summary, generally, we could say that Easterners are less likely to use humor as a coping strategy in comparison with their Western counterparts because of the East-West cultural difference in humor perception. When it comes to the specific humor types, it seems that Easterners tend to use less aggressive but more affiliative humor than Westerners. However, the result is highly contingent on the specific cultural dimensions valued in different cultural backgrounds. It is true when collectivism that stresses harmony and interdependence dominates in Eastern culture. The cultural difference in specific humor usage has yet been conclusive due to a lack of consideration of specific cultural variables. Furthermore, some humor coping strategies may have different connotations under different cultural backgrounds, which would directly impact how humor is used in different cultural backgrounds.

## Relationship Between Cultural Differences in Humor and Psychological Well-Being

Humor has implications for both physical and psychological well-being. Martin (2001) proposed four theoretical mechanisms underlying humor’s effect on health: positive changes in the physiological system brought by laughter, positive emotions accompanying humor, the stress-moderating effect of humor, and the social support associated with humor’s social facilitation function. Martin and Ford (2018) further suggested that the buffering effect of humor comes from cognitive reframing and emotional management.

In fact, psychologists have accumulated evidence for the positive effects of humor: humor improves immunity, facilitates creativity, saves face, relieves stress and tension, creates a more positive self-concept, improves relationships with others, lowers stress, and increases positive emotions and life satisfaction (e.g., Kuiper and Martin, 1993; Martin et al., 1993; Kuiper and Olinger, 1998; Parrish and Quinn, 1999; Lefcourt, 2001; Martin, 2001, 2002; Abel, 2002; Yue et al., 2010a; Cheung and Yue, 2012; Martin and Ford, 2018).

Not all forms of humor are beneficial. As discussed earlier, affiliative, aggressive, self-enhancing, and self-defeating humors are four humor types that seem to exist in both Western and Eastern cultures (Martin et al., 2003; Chen and Martin, 2007). Research on Western cultural background has suggested that affiliative and self-enhancing humors are adaptive, whereas aggressive and self-defeating humor are maladaptive (e.g., Martin et al., 2003; Kuiper et al., 2004). Research has shown that affiliative humor can promote adjustment; relieve anxiety, depression, and attachment avoidance; and increase subjective well-being, individuals’ sense of identity and belongingness (e.g., Chen and Martin, 2007; Cann et al., 2008; Frewen et al., 2008). Self-enhancing humor can help one cope with stress and misfortunes in life; it is positively associated with optimism and self-esteem and negatively associated with depression (Thorson et al., 1997; Chen and Martin, 2007; Dozois et al., 2009; Martin and Ford, 2018). Aggression humor is maladaptive in terms of bringing mental health benefits, as it may deter adjustment and resilience and cause attachment avoidance, lower self-esteem, loneliness, aggression, and maladjustment in the family (e.g., Martin et al., 2003; Kuiper et al., 2004; Kazarian and Martin, 2006; Cann et al., 2008). Self-defeating humor is also detrimental to adjustment, as it may trigger depression or anxiety (e.g., Martin et al., 2003; Chen and Martin, 2007; Cann et al., 2008; Martin and Ford; 2018).

Despite Easterners generally being less likely to use humor as a coping strategy in comparison with their Western counterparts, Easterners also benefit from humor. For example, affiliative and self-enhancing humor could help sojourning mainland Chinese students studying in Hong Kong cope with acculturative stress, increase their level of life satisfaction, and decrease their depressive mood (Cheung and Yue, 2012). It is positively associated with higher levels of self-compassion for Hong Kong students (Yue et al., 2017), higher levels of optimism, and lower levels of loneliness and distress (Sun et al., 2009; Yue et al., 2010a, 2014b; Cheung and Yue, 2013), for both mainland Chinese and Hong Kong students, and is positively associated with self-esteem for mainland Chinese, Hong Kong, and Indian students (Yue and Hiranandani, 2014; Yue et al., 2014a).

Maladaptive humor also seems to be detrimental to Easterners’ psychological well-being, although the results are not that consistent. Consistent with Westerners, self-defeating humor is positively correlated with loneliness and self-esteem for Chinese (Sun et al., 2009; Yue et al., 2014b). Cheung and Yue (2013) found that aggressive and self-defeating humor were positively associated with depression, anxiety, and irritation for Chinese students from Guangzhou, Hong Kong, and Macau. However, when investigating how the four types of humor impact mainland Chinese’s adjustment when studying in Hong Kong, Cheung and Yue (2012) found that aggressive and self-defeating humor did not consistently affect mainland Chinese adjustment. Additionally, Hiranandani and Yue (2014) found that aggressive humor was not significantly associated with self-esteem for Indian or Chinese students. Using Hong Kong students as their sample, Yue et al. (2014a) found that aggressive humor was not significantly associated with self-esteem or subjective well-being.

These inconsistent results may be due to the Chinese dialectic thinking style, which is characterized by contradiction (Peng and Nisbett, 1999; Hou and Zhu, 2002; Ji et al., 2010). Take the inconsistent results of aggressive humor, for instance. On the one hand, Chinese culture emphasizes interdependence and harmony (Hwang, 1987; Cheung et al., 2001), which makes Chinese less likely to use aggressive humor in their daily life (Chen and Martin, 2007; Yue, 2010, 2011). On the other hand, aggressive humor can still buffer harm (Martin and Ford, 2018). The contradictory characteristic of the Chinese thinking style makes Chinese behave in different manners from how they think, thus further blurring the relationship between maladaptive humor and psychological well-being. Alternatively, this may be because the direct translation of the English Humor Style Questionnaire (HSQ) could not fully capture maladaptive humor types in China.

Some research has examined the cultural differences in humor implications systematically. Investigating Chinese and Canadian samples simultaneously, Chen and Martin (2007) did not find cultural differences in the relationship between humor and psychological well-being. A similar correlational pattern was found in both Chinese and Canadian samples. They found that for both Chinese and Canadian students, mental health was positively associated with affiliative and self-enhancing humor but negatively associated with aggressive and self-defeating humor. Furthermore, Cheung and Yue (2012) found that for mainland Chinese students who study in Hong Kong, affiliative humor appeared to buffer all four kinds of hassles linked with depression, whereas self-enhancing humor only buffered the study hassles linked with depression. This indicates that self-enhancement humor is not that potent in helping mainland Chinese to cope with acculturative stress. This may be attributed to Chinese not adopting the Western way of self-enhancement (Sedikides et al., 2003). Yue et al. (2010a) reported that adaptive humor types (affiliative and self-enhancing humor) were positively and maladaptive humor types (aggressive and self-defeating humor) were negatively associated with optimism, for both mainland Chinese and Hong Kong students. The correlation was stronger for mainland Chinese than for the Hong Kong students. Yue et al. (2014b) found that self-defeating humor explained social and emotional loneliness for Hong Kong students but only social loneliness for mainland China students. These results may be because that mainland Chinese students use more affiliative and self-enhancing but less aggressive and self-defeating humor than Hong Kong students (e.g., Yue et al., 2010a, 2014b), as a consequence of the weaker influences of collectivism and Confucianism in Hong Kong society (Yue et al., 2010a).

In sum, Westerners and Easterners do differ in the relationship between humor and psychological well-being, not qualitatively but quantitatively. Although Chinese students tend to appreciate and use humor less than Western students do, Chinese are still more likely to embrace adaptive humor styles, which have the greatest influence on their mental health, while maladaptive humor styles are less influential.

## Discussion and Future Directions

Humor is a pan-cultural phenomenon but is also interculturally different. Previous literature provides substantial evidence suggesting that perceptions of humor, humor usage, and the relationship between humor and psychological well-being differ across cultures.

Specifically, Easterners do not hold a positive attitude toward humor as their Western counterparts do. Among Easterners, Chinese have ambivalent attitudes toward humor. This perception makes Easterners less likely to use humor as a coping strategy in comparison with their Western counterparts. When it comes to the four humor types, Easterners seem to use less aggressive but more affiliative humor than Westerners. However, this result is highly contingent on the specific cultural dimensions valued in different cultural backgrounds. It is true when the Eastern culture that stresses harmony and interdependence. It is not the case when the culture is not characterized by harmony and interdependence. To sum up, we argue that the cultural difference in specific humor usage has not yet been made conclusive due to a lack of consideration of specific cultural variables. Besides, different cultures interpret specific types of humor differently, which influences how humor is used in different cultural backgrounds. Moreover, Westerners and Easterners have a parallel relationship between humor and psychological well-being index. Both Westerners and Easterners benefit from adaptive humor styles and get harm from maladaptive humor styles. However, the strength of the relationship varies across cultures, with maladaptive humor styles are less influential in Eastern cultural background. This may be due to the fact that Easterners are less likely to use maladaptive humor styles, such as aggressive humor (Chen and Martin, 2007). These findings inform the understanding of cultural differences in humor and could assist practitioners in using humor to promote human psychological well-being. However, most of the research has not been conducted systematically and lacks empirical evidence. No causal conclusions can be drawn, and there is still a lot of need for future investigations.

First, most of the research that has been done was largely correlational and based on survey methods. Little research has been conducted to systematically investigate the cultural differences in humor. Future research should employ empirical methods to provide causal evidence for a relationship between humor and culture. In addition to using survey methods to study the differences in humor across cultures, more efforts should be dedicated to investigating how specific culture dimensions influence humor perception, humor usage, and the relationship between humor and psychological well-being across cultures. Besides, empirical research on the relationship between humor and psychological well-being has rarely been conducted; thus, no causality can be drawn. Future research should also employ empirical methods to clarify the causal relationship between humor and psychological well-being. These efforts would help to show the relationship more specifically and systematically.

Furthermore, research on how Eastern culture impacts humor has mostly been conducted within Chinese culture (with participants including mainland Chinese, Hong Kongers, and Taiwanese). To have a comprehensive understanding of how culture influences humor, it would be important to investigate the relationship between culture and humor cross-culturally. Future research should work on examining whether people in other countries under the influence of Confucianism show similar patterns of humor perception, its usage, and its implications on psychological well-being as Chinese do.

Moreover, as we noted above, different cultures have different interpretations of specific humor items. This could explain the inconsistent results about humor across cultures. For example, aggressive humor was not correlated with self-esteem or life satisfaction among Hong Kong participants (Yue et al., 2014a). We suggested that this may be due to the contradiction characteristic of the Chinese thinking style. Alternatively, it also could be due to different interpretations of specific humor items across cultures. Some research (e.g., Cheung and Yue, 2012; Yue et al., 2014a) used direct translations of the English version of the HSQ to tap the four humor types. The items may be not fully reflective of aggressive humor for Chinese. Further research is needed to test these two possible reasons. What’ more important, in the future, researchers should develop a reliable, well-established, having good psychometric property humor style scale within Eastern cultural background instead of direct translation.

Furthermore, the Chinese apprehension–despising complex toward humor needs exploration. The little research on this complex is just descriptive (e.g., Yue, 2010, 2011). The cognitive, emotional, and motivational components of this complex remain unclear, as does how this complex impacts humor usage and the relationship between humor and psychological well-being. We still do not know how other philosophies, such as Buddhism, impact this apprehension–despising complex. All of these questions need future investigation. Besides, different philosophies exist in Western culture (Collins, 2016), and these philosophies could impact how Westerners perceive and use humor, as well as humor and psychological well-being relationship. However, none of these previous research has systematically investigated how different philosophies in Western world influence humor perception, usage and humor’s implications for psychological well-being. Future research should work on this so as to further our understanding of humor in Western cultural background.

Future research should also examine how other possible moderators [such as individual personality traits, political atmosphere, self-esteem, socioeconomic status (SES), context salient needs, etc.] interact with culture in influencing humor perception, usage, and its implications. For example, Westerners with higher self-esteem were more likely to use adaptive humor (Martin et al., 2003). Shy Westerners who were low on perceived social competence tended to use less affiliative humor and more self-defeating humor (Fitts et al., 2009). Will this be true for Easterners as well? Besides these individual differences, one should also consider the contextual factors. For example, people may use humor as a tactic to cope with failure, awkwardness, or face threats (Kane et al., 1977; Cupach and Metts, 1994). Saving face is one of the most important attributes for Chinese (e.g., Gao, 1998; Yue, 2011). Thus, despite Chinese generally being less likely to use humor as a coping strategy, humor becomes a useful tactic for Chinese to adopt when saving face. Chen et al. (2013) found that for Chinese students, affiliative and self-enhancing humor were positively associated with collectivism and the willingness to save one’s own face, and aggressive and self-defeating humor were positively associated with the willingness to save one’s own face. These results indicate that despite their general tendency to use less humor, Chinese like to use humor in coping with face threats. Future research should continue to work along this direction to investigate how other possible individual or situational factors interact with culture in influencing humor perception or usage.

In addition, based on the literature review above, some unique cultural factors may strengthen or weaken the relationship between humor and psychological well-being. However, we do not know what the factors are or how these factors impact the relationship between humor and psychological well-being index. This is worth investigating in future research. For example, it would be interesting to measure mainland Chinese and Hong Kong people’s Confucian views and investigate whether Confucian views could impact the strength of affiliative humor’s effects on optimism.

More importantly, culture is not a static construct. Globalization has brought massive changes in individuals’ psychology and culture. As suggested by Cai et al. (2019), there has been a general increase in individualism and a decrease in collectivism all over the globe. It is certainly true that this change would be manifested in humor, which is worth investigating. Moreover, most of the research on humor and culture has basically been based on comparisons across countries. For example, Saroglou and Scariot (2002) reported that Belgians reported less use of self-enhancing humor and more use of aggressive humor than Canadians. Kazarian and Martin (2004) found that Lebanese reported lower use of self-enhancing humor than Canadians, lower aggressive humor use than Belgians, and lower use of affiliative humor than both Canadians and Belgians. Kalliny et al. (2006) found that Americans scored higher on self-enhancing and self-defeating humor than Arabs. Caution should be taken when drawing conclusions about cultural differences in humor from these research. Without specific cultural dimensions being taken into account, it is hard to conclude that these results could be explained by cultural differences. Besides, globalization has brought about a multicultural context within a geographic location (Cai et al., 2019). Thus, it may not be that accurate to study cultural differences in humor by comparing different nationalities or different geographic locations. Future research should work on studying cultural constructs more precisely. Studying how specific cultural dimensions influence humor perception, humor usage, and the relationship between humor and psychological well-being is one way to solve this problem.

Another possible direction for future research is to specify the meaning of humor under different cultural backgrounds. For example, Liao (2003) differentiated the Chinese term “youmo (direct translation of English humor)” from “huaji (Chinese word for humor since the Spring-Autumn Period)” (p. 1). The results showed that *youmo* is regarded as a high-SES vocal act triggering thoughtful a smile with profundity, while *huaji* is described as low-SES, funny, shallow, and ridiculous actions that trigger more laughter than smiles with ridiculousness. One may expect Chinese to use *youmo* and *huaji* differently and that the usages will have different implications for Chinese psychological well-being. What’s more, the different perception of humor as *youmo* and *huaji* might be associated with different philosophies in Chinese culture. Specially, Confucianism devalues humor (Liao, 1998, 2007; Yue, 2010), which may make people lack of humor or demonstrate humor in a decent and sophisticated way. On contrary, people who believe in Taoism would like to tell witty jokes (Yue, 2011), which may make them prefer *huaji* to *youmo*. Future research should devote efforts to examine these possibilities.

## Conclusion

Humor is an adaptive strategy with which individuals can survive severe competition (Wilson et al., 1977; Martin and Ford, 2018). No societies or groups have been found having no sense of humor (Fry, 1994). In this article, we reviewed how humor perceptions, humor usage, and the relationship between humor and psychological well-being index differed across cultures. We also discussed some limitations in this area that need research. Understanding how people view and use humor, how humor impacts psychological well-being, and how culture influences it is an emerging area of empirical research that promises to be an important area worth future investigation. More attempts should be made to better understand the relationship between humor and culture as a way of promoting better psychological well-being.

## Author Contributions

TJ and YH conceptualized the manuscript. TJ wrote the first complete draft. YH and HL contributed additional writing. All authors edited the manuscript and approved the final version.

## Conflict of Interest Statement

The authors declare that the research was conducted in the absence of any commercial or financial relationships that could be construed as a potential conflict of interest.
